# Grouper (Epinephelidae) spawning aggregations affect activity space of grey reef sharks, *Carcharhinus amblyrhynchos*, in Pohnpei, Micronesia

**DOI:** 10.1371/journal.pone.0221589

**Published:** 2019-08-28

**Authors:** Kevin L. Rhodes, Ivy Baremore, Rachel T. Graham

**Affiliations:** 1 MarAlliance, San Francisco, CA, United States of America; 2 Pacific Marine Science and Conservation, Grass Valley, CA, United States of America; Institut de recherche pour le developpement, FRANCE

## Abstract

Fish spawning aggregations (FSA) act as biological hotspots that concentrate food and nutrients across a broad trophic spectrum. In Pohnpei (Federated States of Micronesia), 20 female grey reef sharks (*Carcharhinus amblyrhynchos*) were acoustically tagged at two multi-species grouper (Epinephelidae) FSA to examine the likelihood that these mesopredators utilize FSA as a seasonal food source. Both FSA sites are within small-scale MPAs, thus providing a secondary opportunity to examine their conservation potential during these ephemeral events. Shark movement and residency was gauged against known spatial and temporal grouper reproductive patterns using an array of 15 and 50 acoustic receivers at Ant Atoll and Pohnpei (Island), respectively. Activity space was investigated using Kernel Density estimates of individual sharks, and residency indices (RI) were analyzed based on daily and monthly occurrence at the array. Three distinct residency patterns were identified: transient, semi-transient, or resident (Daily RI <0.40, >0.40 to 0.80, or >0.80, respectively). Generalized linear mixed models (GLMMs) were used to identify biological and environmental factors influencing shark activity space, including month, temperature, shark size, spawning month, and residency pattern. Findings revealed significant changes in average monthly residency indices and kernel densities during spawning months in support of an opportunistic foraging strategy around FSA. Monthly residency was higher during spawning months among semi-resident and transient sharks, while average monthly activity space was concentrated around FSA. Best-fit models for the GLMM indicated that activity spaces were most influenced by month and grouper spawning month. Seven of 20 sharks demonstrated inter-island movement and wide variations in individual movement and spatial requirements were shown. The concentration of sharks and groupers at unprotected FSA sites increases their vulnerability to fishing and supports the need for combined area and non-area management measures to effectively protect these species.

## Introduction

Fish spawning aggregations (FSA) represent critical life history phases for a number of coral reef fishes [1] and serve as biological hotspots that provide food and nutrients to marine organisms across a wide trophic spectrum [2–7]. In many locales, FSA are multi-species and may be comprised of 100s or 1000s of individuals [8–10], thus representing a rapid and substantial increase in biomass and nutrient flow within areas used as spawning sites. Since FSA are spatially and temporally predictable, they are highly attractive to both fishers who can extract high catch volumes over brief time periods [11–13] and to marine organisms that gain from the elevated food and nutrients available during these events [14]. For fisheries, the benefits of FSA fishing are well known [13, 15] as are the impacts that targeted FSA fishing can have on stocks [16, 17], particularly when FSA remain unprotected. In extreme instances, aggregation fishing can fully extirpate FSA [18], with concomitant losses in food and nutrients to the organisms reliant on them.

Among the many species benefitting from FSA through foraging are sharks and rays, with perhaps the most spectacular foraging events associated with whale sharks (*Rhincodon typus*) [4, 19–22] and reef-associated mantas (e.g. Alfred manta, *Mobula alfredi*) [23]. Less observed, but equally spectacular are the foraging events on FSA by subtropical and tropical reef-associated requiem sharks (Carcharhinidae), including lemon sharks (*Negaprion brevirostris*; Poey 1868) [24], grey reef sharks (*Carcharhinus amblyrhynchos*; Bleeker 1856) [14, 25], and blacktip reef sharks (*Carcharhinus melanopterus*; Quoy and Gaimard 1824) [26]. During these periods, sharks may become vulnerable to the same types of gear used for target aggregating fish (Rhodes pers. observ.), thus protective measures that target spawning fishes may also provide a temporary respite from fishing for sharks.

In the tropics, there is a drive to establish marine protected areas (MPAs) as a means to protect reef-associated marine resources. Similarly, the International Marine Protected Area Congress seeks to designate 10% of the world’s oceans as MPAs by 2020 [27]. Thus, the need to examine the utility and optimal design of these set-asides for highly mobile and migratory organisms, such as transient spawners and reef-associated sharks is imperative. For transient FSA-forming species, a number of conventional and acoustic telemetry studies has been conducted to examine seasonal and monthly movement patterns, residency, visitation frequency and fishing vulnerability [28–33]. Those studies largely confirm the utility of MPAs in protecting aggregating fish, but point to the need for additional management measures to protect migrating fish and fish using aggregation sites. Similarly, numerous tagging studies have focused on reef-associated and pelagic shark movement, both to assess MPA effectiveness and identify other appropriate conservation measures [24, 34–38].

Among coastal sharks, grey reef sharks are one of the most prominent tropical reef-associated Indo-Pacific species. Several studies have investigated their movement [34–38] and the potential benefits of small-scale MPAs (herein ≤1 to 5 km^2^). In general, findings have shown sex-specific differences in habitat preferences and movement patterns and distances, with females generally less likely to undergo long-distance movement than males. Grey reef sharks also tend to be more resident when reefs are continuous or closely spaced [37]. Where reefs are semi-isolated, grey reef sharks appear to be largely resident with some long-distance movement possible [38–40]. In contrast, in isolated reef settings, grey reef shark residency can be high [36], but movements away from isolated habitats may also be substantial, covering hundreds of kilometers [41].

In the western and central Pacific, the Micronesia Challenge was initiated in 2005 with the primary goal of incorporating 30% of available nearshore marine habitat into marine reserves (http://micronesiachallenge.org). While there has been success in achieving this goal, most reserves are small (<10 km^2^), nearly all are unenforced and only a few have been placed in areas of biological significance, i.e. critical habitat or areas of high biodiversity. Indeed, a recent assessment showed a limited efficacy of MPAs in the region [42]. Currently, it is unclear whether the intended benefits of these reserves will be realized, particularly for mobile animals, such as reef-associated sharks and aggregating groupers [43]. Questions about the efficacy of MPAs for sharks have arisen based on empirical evidence. For example, in the Great Barrier Reef grey reef sharks have continued to experience large-scale declines (17% yr^-1^) in spite of relatively strict enforcement in four types of management zones that include no-entry reserves [44]. In contrast, subsequent fishery-dependent findings [45] reported that current management for sharks was effective, although the majority of shark by-catch was grey reef sharks.

Similar to the use of MPAs for sharks, the results of small-scale MPAs for FSA have been mixed. In Pohnpei, Micronesia, year-over-year declines in fish density have continued inside a small-scale MPA meant to protect spawning grouper [10]. In contrast, improvements in fish density have been shown for MPA-protected FSA in Papua New Guinea [46]. In the Caribbean, improvements in FSA abundance and size were demonstrated following combined area and harvest measures for red hind (*Epinephelus guttatus*) [47]. Clearly, there is a need for greater understanding of habitat use, movement and vulnerability of these mobile organisms.

Although grey reef sharks are among the most common reef-associated mesopredators, they are also among the most vulnerable to small-scale fisheries. Grey reef sharks were listed by the International Union for the Conservation of Nature (IUCN) as Near Threatened (NT) during the last global evaluation in 2005. Population trends at that time were listed as unknown [48]. In recent years, declines in grey reef sharks have been associated with targeted and non-targeted fisheries that are drawn to the commercial value of dried fins, meat and teeth. In many small-scale commercial fisheries in the tropics, grey reef sharks are commonly taken as by-catch [49] and often killed and discarded because of the perception by some fishers that they impede fishing success (Rhodes pers. observ.). Among larger scale commercial fisheries, passive sexual selection of sharks has been reported from longline fishing [50–51], due in part to segregation by depth in some shark species [52]. Moreover, high site fidelity and philopatry have also been identified among coastal shark species [53–55], suggesting localized depletions are possible where sharks are concentrated and remain unprotected. As a result of these various shark fisheries and observed sex-specific behavioral differences [56–57], populations in some locales have been severely depleted [58]. Even with fisheries management in place, grey reef sharks may be vulnerable to fishing mortality as they have a moderate rebound potential, with gestation over 9-months, litter size ranging from 3–6 pups, and a mean of 4.1 pups (e.g. Hawaii) [59].

In Pohnpei (Federated States of Micronesia, FSM hereafter), populations of all nearshore shark species are anecdotally reported as declining (Rhodes, pers. observ.). Local fisheries for sharks include the capture of juveniles for teeth and jaws for the curio trade, however on the main island and atolls immediately surrounding Pohnpei (Island), there is no targeted food fishery for sharks. Small-scale commercial and subsistence fishers view sharks of all kinds as a nuisance and are known to remove the tail and discard the carcass when landed (Rhodes, pers observ.). Some small-scale shark finning is known to occur within the artisanal fishery. Further investigation is needed to gauge the scale and impact of the existing fishery, notably in light of the ban on shark fishing in Pohnpei’s territorial waters enacted by the FSM Government in 2013. The national ban was expanded as part of the declaration of the Regional Micronesia Shark Sanctuary in 2015, which encompasses 6.5 million square kilometers, and where the region’s governments prohibit the commercial fishing of sharks, retention of sharks caught as by-catch, and the trade, possession, and sale of shark products. The impact of the sanctuary on a reversal in declines of shark populations has not been evaluated.

**Fig 1:**
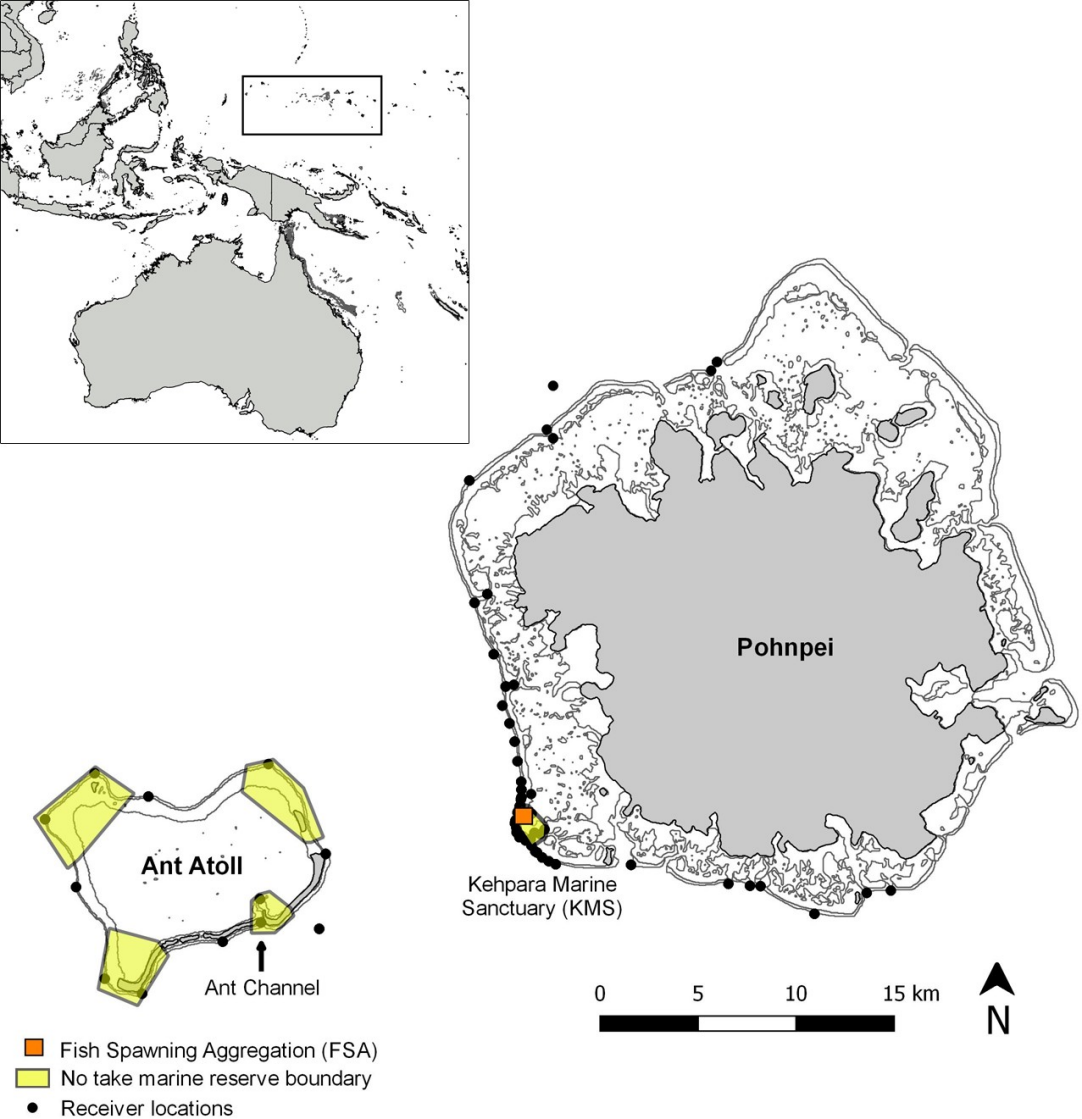
Map of the study site. Pohnpei and Ant Atoll, Federated States of Micronesia showing receiver locations. Boxes with yellow shading indicate the boundaries of no-take marine reserves and land masses are shaded grey. Orange boxes denote fish spawning aggregation (FSA) sites.

Within the state, there are currently 16 small-scale no-take marine reserves ranging in coverage from 0.34 km^2^ to 41.4 km^2^ (mean = 7.4 km^2^; median = 2.1 km^2^); however, most are poorly enforced [10, 60], including the Kehpara Marine Sanctuary (KMS) (Fig 1). The KMS was established in 1999 to protect seasonal spawning aggregations of camouflage grouper, *Epinephelus polyphekadion* (Bleeker 1849; VU A2bd), brown-marbled grouper, *Epinephelus fuscoguttatus* (Forsskål 1775; VU A2bd+4bd), and squaretail coralgrouper, *Plectropomus areolatus* (Rüppell 1830; VU A2bd) (http://www.iucnredlist.org).

Four sections of nearby Ant Atoll were also designated for marine area protection by Pohnpei State in 2007 (Fig 1) and in 2008 the entirety of Ant (34 km^2^) became a UNESCO Man and Biosphere Reserve (http://www.unesco.org). The lagoon and channel are now regularly patrolled by private enforcement. Within the KMS, decadal declines in the FSAs of all three aggregating groupers have been associated with fishing, following a relaxation in enforcement [10]. There has been no baseline assessment of aggregating groupers at Ant or of sharks at any location in Pohnpei.

Within both the KMS and the main channel of Ant Atoll, seasonal (January-May) FSA of these aforementioned three groupers form *c*. two weeks prior to each full moon [35]. Aggregation abundance and density increases daily for all species prior to spawning and the arrival time and duration of individuals varies by sex [61–63]. Spawning occurs on the last day or days of the FSA for each respective species, after which time individuals disperse to home reef areas. Each aggregation typically numbers in the 100s to 1,000s of individuals. At Ant, a number of other teleosts also aggregate during these months, creating an additional attraction to fishers and possibly sharks. Sharks have always been present at the Kehpara and Ant FSA during past research studies [63] and were often captured and released, however it is unclear whether this presence simply represented resident grey reef sharks or whether there was an attraction to the site for trophic opportunism.

The objectives of the current study were to:

- examine monthly activity spaces, as kernel densities, residency and timing of grey reef shark movement in order to discern whether there are significant differences between grouper non-spawning and spawning periods to imply attraction to the site for trophic interactions, and;
- identify what level of protection the small-scale MPAs that characterize much of the developing Pacific, including the existing MPAs at Kehpara and Ant Atoll, might provide for grey reef sharks times when they are concentrated, e.g. during grouper reproductive periods.

## Materials and methods

### Site description

Between 22 and 26 January 2010 and 31 January to 16 March 2012, grey reef sharks were captured at two separate seasonal (transient) grouper spawning aggregation sites each located within small-scale MPAs (Fig 1). In Pohnpei, the KMS was established by a private landowner as a permanent no-take zone in 1995 and formalized by Pohnpei State in 1999. The KMS encompasses a well-studied multi-species grouper FSA site that has been described in detail in previous reports [35, 39–41]. The KMS is *ca*. 1.5 km^2^ and includes both inner and outer reef habitat along the southwestern portion of Pohnpei (Kitti Municipality). Within the KMS, groupers aggregate monthly on the outer reef wall over an approximate 2-week period prior to full moon between January and May: *P*. *areolatus—*January-May; *E*. *fuscoguttatus*—February-May; *E*. *polyphekadion*—February-March or March-April. The initiation of each species’ spawning season and the month of peak density are all dependent on the timing of full moon relative to winter solstice [10]. In this way, the reproductive season for *E*. *polyphekadion*, for example, can be either February-March or March-April. For *P*. *areolatus*, there are indications the FSA in January represents an all-male aggregation that starts around new moon and remains in place over a 6-week period [62]. In subsequent months, male *P*. *areolatus* depart along with females following spawning around the full moon. All three species have been shown or observed utilizing common reproductive migratory corridors to reach and depart the FSA site and at last some individuals travel up to 25 km from the FSA to reach home reefs immediately after spawning [62–64]. As previously indicated, the species-specific duration of FSA varies, with *P*. *areolatus* and *E*. *fuscoguttatus* maintaining aggregations over *c*. 2 weeks during each reproductive month, while the *E*. *polyphekadion* FSA currently extends over 5 days or less [10]. This truncated aggregation period is considered an effect of overfishing and represents a decline from two decades earlier when these FSA formed and persisted for 10–12 days [61]. Depending on the timing of seasonal aggregation formation, February, March and April are peak aggregation months in terms of abundance, and months where either two or all three species can be present.

Prior acoustic telemetry studies and fishery-independent surveys for grouper at the KMS showed that grey reef sharks inhabit the area and readily prey on line-caught groupers during retrieval. Enforcement at KMS occurred from the time of its designation as an MPA until 2005 when the private landowner died. Since that time, poaching has been ongoing, with year-over-year declines in grouper abundance and density [10]. Another FSA site, located in the channel of neighboring Ant Atoll was chosen as the second location for grey reef shark tagging. Unlike Kehpara, the Ant FSA was largely unprotected and actively fished until 2015, particularly during grouper spawning times. Ant Sanctuary was established as a no-take zone in 2007 and is comprised of four sections totally 34 km^2^, however only about one-quarter of the total area is dedicated to the channel where the FSA forms (Fig 1). The site is currently being enforced by private individuals residing on the atoll.

### Tagging

For tagging, grey reef sharks were captured by traditional fishers fishing on snorkel. Fishing was conducted in open water seaward of the reef. Fishers used standard 80-lb clear monofilament tuna line fitted with braided 1/16” stainless steel wire leader and a swivel attached to a 16/0 gauge circle hook. Fishing was conducted during daylight hours using various baits, including grouper, skipjack (*Katsuwonus pelamis*) or kawakawa (*Euthynnus affinis*). Once captured, grey reef sharks were brought to the surface, held boatside on the fishing line to allow water movement across the gills, immobilized using a tail rope and subsequently inverted to induce tonic immobility and facilitate tag implantation. Each captured shark was then measured across the body length in a curved line to the nearest cm pre-caudal (PCL) and total length (TL) [65], and sexed visually prior to surgery. For implantation of acoustic tags (Vemco V16-6L coded acoustic transmitter, 69 kHz, 90-sec blanking interval, estimated tag life = 1877 d) (Amirix Systems, Nova Scotia, Canada), a *c*. 4–5 cm incision into the peritoneal cavity was made using a sterile #10 surgical scalpel blade. Prior to insertion, tags were coated with over-the-counter triple antibiotic ointment. Following insertion, incisions were closed using Ethicon 5–0 cutting edge needle and braided silk sutures. Tagging procedures typically lasted *c*. 8 to 10 min. All fish were checked to ensure they were healthy and active prior to release at or near the point of capture. Of the nine tags deployed in 2010, one was recovered from an animal previously tagged in a separate study and had a maximum of 1411 days remaining battery life. Twelve new tags were implanted in grey reef sharks in 2012, bringing the study total to 21 animals. Pohnpei State Office of Fisheries and Aquaculture reviewed and approved the protocols and provided oversight of the research.

### Acoustic monitoring

Between January 2010 and October 2013, monitoring was conducted on tagged grey reef sharks using 65 Vemco VR2W receivers that were moored along the outer barrier reefs and channels leading into Ant and Pohnpei lagoons (Fig 1). A concentration of receivers was placed within and adjacent to the KMS as part of a separate, ongoing grouper tagging study. Otherwise, receivers were placed north and south of the FSA at *c*. 4-km intervals. The receivers were placed to simultaneously research grey reef sharks and aggregating groupers [63] and covered *c*. 80 km of combined reef area. All receivers were suspended in the water column at *c*. 15–20 m using a hard plastic sub-surface float. All receivers were downloaded and maintained at 1-year intervals or less, depending on opportunity and need. At Ant Atoll, 15 receivers remained deployed along 45 km of outer barrier reef, inclusive of two receivers along the main channel leading into the lagoon (Fig 1). Prior range testing showed that detections of 250 m were common in unobstructed areas [64], similar to that which characterizes the outer reef mooring sites.

### Data analysis

Total detections at each receiver within the array were examined for the study period. Detections at Pohnpei and Ant Atoll were examined by day and month to examine changes in shark presence over time and determine activity spaces. Daily detections (shark detected at the array at least twice during one 24-hr period) of individual sharks were calculated to monitor presence of the sharks within the receiver array and at the FSA, and to assess changes in detection frequency during grouper spawning months. Residency of sharks (residency index = RI) was calculated as the total number of days detected divided by the total number of days the shark was expected at the array if it were detected every day (date array removed-date tagged) [66]. RI was also calculated for days spent within the FSA and the KMS, and during new and full moon cycles. Sharks were considered “resident” when overall RI > 0.80, “semi-transient” when RI > 0.40 and < 0.80, and “transient” when RI < 0.40, similar to the ‘cut-off’ values used by other recent researchers [14]. A monthly RI was also calculated for each shark to determine how individuals’ residency patterns changed over the course of the year. Monthly RI was plotted separately for the resident groups to assess whether these patterns differed among transient, semi-transient, and resident sharks at Ant Atoll and Pohnpei. Differences in RI by month and transient group were examined using Analysis of Variance (ANOVA) if data were determined to be normally distributed and homogenous, otherwise a Kruskal-Wallis test was applied [67]. Monthly RI differences between grouper spawning months and non-spawning months were tested using a Student’s t-test. Differences in mean daily RI between new and full moon periods at the FSA were also tested using a Student’s t-test. Lunar phases were determined using the R package Lunar [68].

The utilization distribution (UD) of individual sharks was examined using fixed Kernel Density (KD) analyses of detections for each individual at 30-minute time steps using a bivariate normal kernel function [69]. The KD calculation uses a kernel method to estimate the KD using relocation (detection) data [70], and a smoothing parameter was chosen based on visual choice after several trials. Core activity space (50% KD) and the activity space extent (95% KD) were examined for changes in habitat use over the course of 46 months. Sequential monthly KD values were calculated for each individual; in instances where the number of detections was insufficient to calculate KD, the individual was omitted from analysis for that month. All calculations and KD were made using the adehabitatHR and sp packages in R [69, 71–72], and visualizations for KD were made in QGIS.

Generalized linear mixed models (GLMMs) were used to examine the monthly changes in activity spaces of sharks in relation to seasonal (January—May) grouper spawning aggregations and other factors, such as water temperature, shark size (TL), and residency status of individuals (transient, semi-transient, resident). Individual (transmitter code) was included in the model as a random factor to account for repeated-measures [73]. Estimates of KD were tested for normality and square root transformed, if necessary [74], and models were checked for multicollinearity by calculating variance inflation factors (VIF). Final model selection was based on Akaike Information Criteria (AIC) [73, 75], and the dredge function [76] (package MuMIn) was used to generate a suite of models for comparison [77]. Models were built with all combinations of factors for 50% and 95% KD (global model: KD ~ month + spawning month + temperature + TL + residency status) and were tested against the null model using maximum likelihood. Model averaging on the top candidate models was conducted: parameter estimates were averaged for a subset of models where deltaAIC < 2, and cumulative AIC weights (w_i_) were used to determine the influence of each factor on activity space, with w_i_ > 0.5 considered significant drivers of activity space [73, 77]. Month was a sequential factor and spawning months were binary (spawning months = January through the end of May of each year), based on previously published information [10]. Temperature was a calculated monthly average based on data loggers that were concurrently placed at the study area.

Differences in monthly estimated 50% and 95% KD were analyzed using a non-parametric Kruskal-Wallis test because the data were non-normal and non-homogenous [67]. In order to assess individual variation in monthly activity space use, Kruskal-Wallis tests were also run separately for monthly KD estimates by residency status (transient, semi-transient, and resident) and plotted. A post-hoc pair-wise Wilcox test was run if differences were significant to determine where differences occurred. A student’s t-test was run to assess differences in activity spaces between grouper spawning months and non-spawning months.

## Results

### Spatial habitat use and residency

Between 22 and 26 January 2010 and 31 January to 16 March 2012, a total of 21 grey reef sharks were captured and fitted internally with an acoustic transmitter, which included four sharks inside Ant Channel and the remainder of sharks within the KMS (Table 1). All individuals were adult females as determined by a combination of size and the absence of claspers. Adult females ranged in size from 101 to 160 cm TL (mean = 147.0 cm TL). The smallest grey reef shark (101 cm TL) tagged at Ant (Transmitter 51334) was detected at the array for only a single day and was removed from the analysis. Among the 20 remaining individuals, total detection days ranged from 250–1347 d (Table 1).

**Table 1:** Tagging information, residency indices (RI), and Kernel Density (KD) estimates^1^.

Transmitter	Date tagged	Location tagged	TL	Tag life (days)	Days monitored	Days detected	Overall RI	RI inside KMS	Overall 50% KD (km^2^)	Overall 95% KD (km^2^)
48007	2-Feb-12	Kehpara MPA	147.5	1877	626	250	0.40	0.29	0.05	0.36
53832	23-Jan-10	Kehpara MPA	152	1850	1366	501	0.37	0.28	2.22	17.87
53838	26-Jan-10	Kehpara MPA	150	1850	1363	321	0.24	0.24	0.19	1.27
53840	22-Jan-10	Kehpara MPA	147	1850	1367	466	0.34	0.33	0.41	2.72
65071	26-Jan-10	Kehpara MPA	150	3650	1363	253	0.19	0.04	2.44	14.16
65075	26-Jan-10	Kehpara MPA	156	3650	1363	298	0.22	0.14	0.37	2.3
7785*	22-Jan-10	Kehpara MPA	158	1411	1411	909	0.64	0.63	0.38	1.82
48006*	28-Jan-12	Kehpara MPA	107	1877	631	432	0.68	0.68	0.13	0.89
48008*	3-Feb-12	Kehpara MPA	151	1877	625	471	0.75	0.46	4.39	43.65
51335*	6-Mar-12	Kehpara MPA	154	1870	593	458	0.77	0.03	2.81	17.83
48010*	31-Jan-12	Ant Channel	146	1877	628	261	0.42	0.03	9.41	109.02
51336*	5-Mar-12	Ant Channel	140	1870	594	435	0.73		0.25	1.92
51337*	4-Mar-12	Ant Channel	152	1870	595	428	0.72		2.39	24.92
65072*	26-Jan-10	Kehpara MPA	148.5	3650	1363	642	0.47	0.24	0.32	2.82
51328**	3-Mar-12	Kehpara MPA	154	1870	596	582	0.98	0.97	0.01	0.07
51329**	3-Mar-12	Kehpara MPA	154	1870	596	585	0.98	0.53	1.22	7.47
51338**	3-Mar-12	Kehpara MPA	156	1870	596	590	0.99	0.98	0.02	0.15
51339**	3-Mar-12	Kehpara MPA	153	1870	596	596	1.00	0.99	0.008	0.06
65074**	26-Jan-10	Kehpara MPA	160	3650	1363	1347	0.99	0.94	0.05	0.79
65076**	26-Jan-10	Kehpara MPA	151	3650	1363	1272	0.93	0.90	0.01	0.88

1 One asterisk indicates semi-transient sharks, two asterisks denote resident sharks, and those with no asterisks are transient.

Tagging information, residency indices (RI), and Kernel Density (KD) estimates of mature female *Carcharhinus amblyrhynchos* tagged with internal transmitters at Pohnpei and Ant Atoll from January 2010-March 2012. Sharks were monitored from January 2010-October 2013.

Residency indices at the array by daily occurrence were high for all sharks combined, with an average (±SD, hereafter) RI of 0.64±0.29 (Table 1). Of the 20 sharks used in the analysis, six individuals were considered transient, eight were semi-transient and six were highly resident (Table 1). All sharks tagged at Ant were considered semi-transients (Table 1).

**Fig 2:**
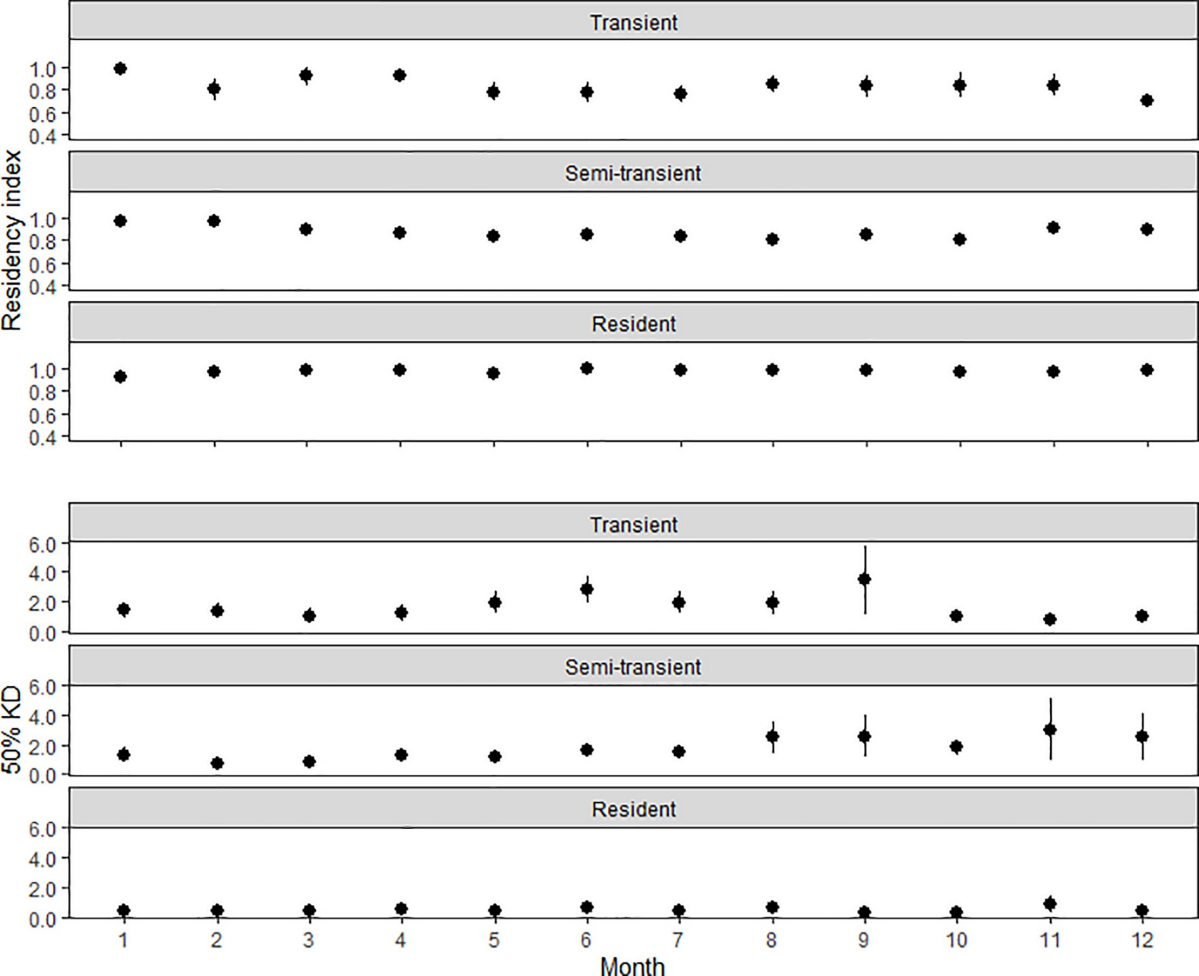
Kernel density and residency index estimates. Monthly 50% and 95% kernel density utilization (KD) and residency index (RI) estimates for all female grey reef sharks *Carcharhinus amblyrhynchos* monitored by acoustic array at Pohnpei and Ant Atoll from January 2010-October 2013. Error bars represent standard error. The outlined box represents the aggregation months.

**Fig 3:**
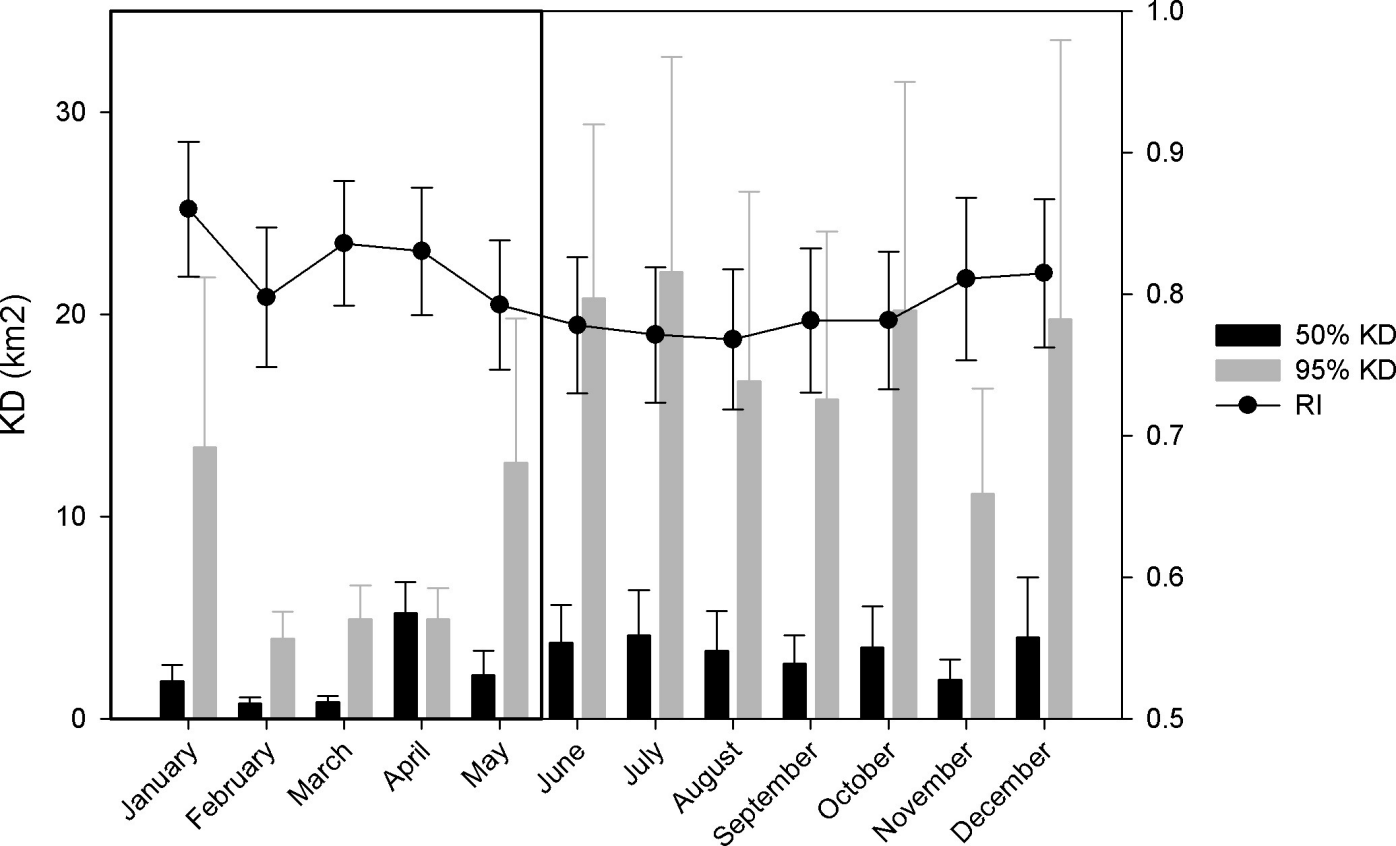
Monthly residency index and kernel density. (A) Monthly residency index (number of days detected divided by the number of days in each month) and (B) Monthly KD for transient, semi-transient, and resident female grey reef sharks tagged at Pohnpei and Ant Atoll, Micronesia during two tagging events in 2010 and 2012. The black box outlines aggregation months (January–May), while the shaded area represents months when either two or all three groupers are present (February–April).

The number of days sharks were detected within the KMS was similar to those spent outside the KMS (8,103 days inside vs. 7,439 outside; 64% vs. 71% of total days each shark was detected at the array). Two of the 20 sharks were never detected at the KMS, both of which were initially tagged at Ant. Residency within the KMS was highly variable, with an average overall RI of 0.48±0.36. Sharks that were considered transient at the array spent the least amount of time within the KMS (RI = 0.22±0.11), followed by semi-transient sharks (RI = 0.34±0.29) (Table 1). Of six sharks considered to be resident to the array, five had a daily RI ≥ 0.90±0.17 within the KMS (Table 1). The 18 sharks detected at the FSA throughout the study had an average daily RI of 0.47±0.35. The daily RI was not significantly different at the FSA between full moon and new moon periods (Student’s t-test, p = 0.37). Average monthly RI at the array for all sharks was not significantly different across all months (ANOVA, p>0.05), with an average of 0.80±0.30 (Fig 2). There were insignificant trends (ANOVA, p>0.05) in decreasing average monthly RI among semi-transient and transient sharks, while resident sharks maintained similarly high RI across all months (Fig 3A). Monthly average RI was significantly higher at the FSA during spawning months versus non-spawning months for all residency types (0.59±0.38 vs. 0.52±0.37, respectively; Student’s t-test, p<0.05).

### Activity space

**Fig 4:**
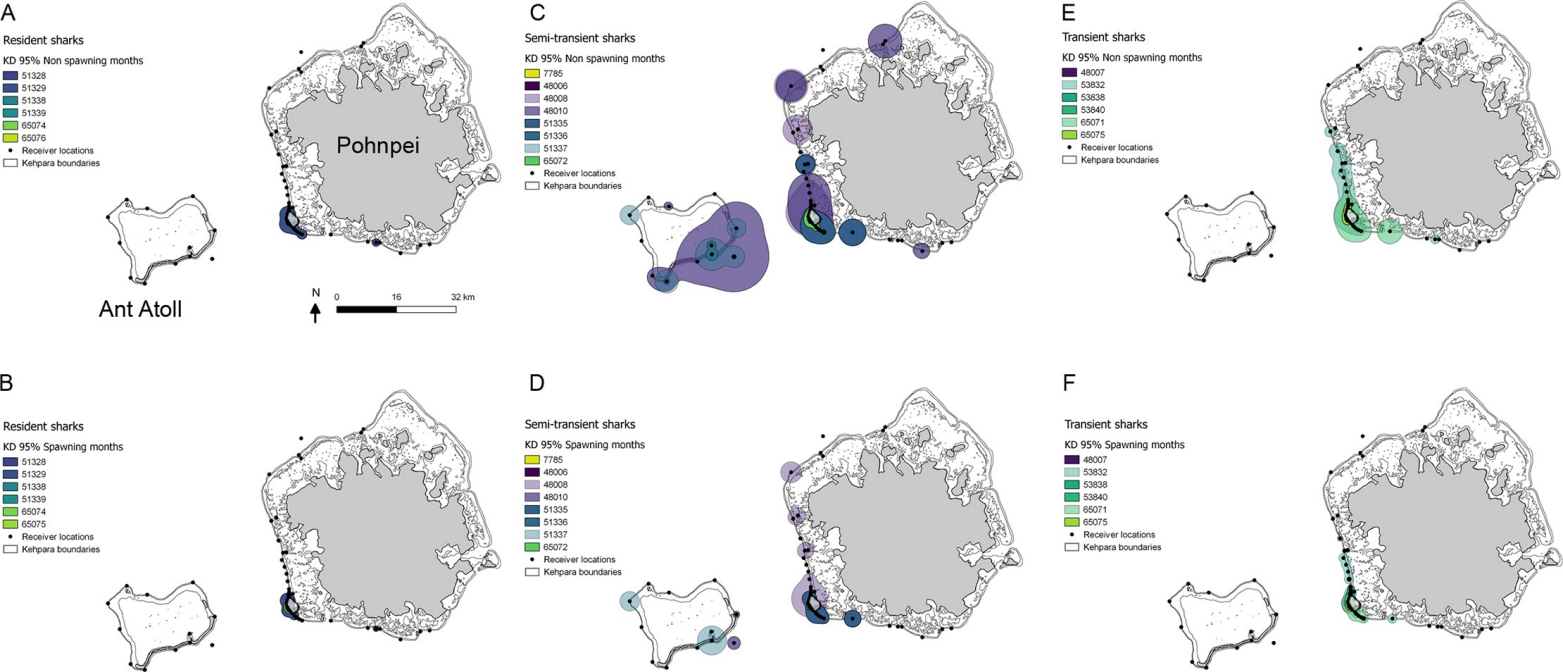
Shark activity space. Activity spaces for 20 female grey reef sharks calculated as 95% Kernel density (KD) utilization for A) resident sharks overall; B) resident sharks during grouper spawning months; C) semi-transient sharks overall; D) semi-transient sharks during grouper spawning months; E) transient sharks overall; and F) transient sharks during grouper spawning months.

Large variations in activity space (KD) were observed among individuals. By residency type, semi-transient and transient sharks showed a much wider range of movement and activity space outside of the aggregation periods (Fig 3B). Activity spaces were small overall, with only one shark utilizing both Pohnpei and Ant Atoll regularly (Transmitter 48010, 95% KD = 109.0 km^2^). The extent of activity spaces of all other sharks was < 45 km^2^ (95% KD; Fig 4; Table 1) and most were under 20 km^2^. The semi-transient sharks had the largest average activity spaces (mean 50% KD = 2.1 km^2^, 95% KD = 20.6 km^2^), while the resident sharks had the smallest (Table 1). The six resident sharks at the array had an average activity space core of just 0.32 km^2^ (50% KD) and an extent of 2.1 km^2^ (95% KD). All resident sharks spent a high number of days within the KMS (Table 1).

In general, activity spaces increased over the course of the year, although there were not significant differences detected among months (averaged by calendar month) (Kruskal-Wallis p > 0.05, Fig 2). Overall, activity spaces for resident sharks remained constant, while non-significant increases were observed among semi-transient and transient sharks for the core (50% KD) (Fig 3B) and extent (95% KD) of activity spaces. Average monthly 50% KD and 95% KD estimates were significantly lower during grouper spawning months than non-spawning months (2.5 vs. 7.3 km^2^ and 7.8 vs. 38.8 km^2^, respectively; Student’s t-test, p < 0.05) (Figs 2 and 4).

Results for mixed models indicated that month and grouper spawning period influenced the activity space of sharks at the study site (Tables 2 and 3). The best-fit model for core activity space (50% KD) (Table 2) included month and grouper spawning period, and the best-fit model for the extent of activity space (95% KD) (Table 3) included only month. All candidate models were significantly different than the null model (p < 0.001). Model results were similar between the 50% and 95% KD estimates, with the top three models for both scenarios including month, grouper spawning period, and residency status (Tables 2 and 3). AIC values were similar for the top candidate models, indicating weak support.

**Table 2:** The 15 top-ranked GLMMs for the 50% Kernel Density (KD) activity spaces.

Model 50% KD	df	Loglik	AIC	deltaAIC	w
**Month+Spawning**	**5**	**-751.55**	**1513.30**	**0.00**	**0.22**
Month	4	-753.01	1514.10	0.88	0.14
Month+Spawning+Resident	6	-751.10	1514.40	1.18	0.12
Month+Temp	5	-752.32	1514.80	1.54	0.10
Month+Spawning+TL	6	-751.47	1515.20	1.92	0.08
Month+Spawning+Temp	6	-751.50	1515.20	1.98	0.08
Month+Resident	5	-752.65	1515.50	2.21	0.07
Month+Resident+Temp	6	-751.84	1515.90	2.66	0.06
Month+TL	5	-752.92	1516.00	2.74	0.06
Month+Temp+TL	6	-752.24	1516.70	3.45	0.04
Month+Resident+TL	6	-752.61	1517.50	4.19	0.03
Spawning+Resident	5	-756.65	1523.50	10.20	0.00
Spawning	4	-757.76	1523.60	10.36	0.00
Spawning+Resident+Temp	6	-756.01	1524.30	11.00	0.00
Spawning+Temp	5	-757.21	1524.60	11.32	0.00

Results from the 15 top-ranked GLMMs for the 50% Kernel Density (KD) activity spaces of female grey reef sharks *Carcharhinus amblyrhynchos* at Pohpei and Ant Atoll from January 2010-October 2013. The best-fit model with the lowest AIC is shown in bold. Month: sequential month from the start of the study; Spawning: grouper spawning period (binary); Resident: residency status (transient, semi-transient, resident); Temp: temperature; TL: total length.

**Table 3:** The 15 top-ranked GLMMs for the 95% Kernel Density (KD) activity spaces.

Model 95% KD	df	Loglik	AIC	deltaAIC	w
**Month**	**4**	**-877.39**	**1762.90**	**0.00**	**0.20**
Month+Spawning	5	-876.43	1763.00	0.15	0.19
Month+Spawning+Temp	6	-875.93	1764.10	1.22	0.11
Month+Resident	5	-877.11	1764.40	1.51	0.09
Month+Spawning+Resident	6	-876.09	1764.40	1.53	0.09
Month+TL	5	-877.37	1764.90	2.03	0.07
Month+Temp	5	-877.38	1764.90	2.05	0.07
Month+Spawning+TL	6	-876.42	1765.10	2.20	0.07
Month+Resident+TL	6	-877.11	1766.50	3.58	0.03
Month+Temp+Resident	6	-877.11	1766.50	3.58	0.03
Month+Temp+TL	6	-877.37	1767.00	4.10	0.03
Spawning	4	-881.41	1770.90	8.05	0.00
Spawning+Resident	5	-880.51	1771.20	8.30	0.00
Spawning+Temp	5	-881.36	1772.90	10.01	0.00
Spawning+TL	5	-881.41	1773.00	10.10	0.00

Results from the 15 top-ranked GLMMs for the 95% Kernel Density (KD) activity spaces of female grey reef sharks *Carcharhinus amblyrhynchos* at Pohpei and Ant Atoll from January 2010-October 2013. The best-fit model with the lowest AIC is shown in bold. Month: sequential month from the start of the study; Spawning: grouper spawning period (binary); Resident: residency status (transient, semi-transient, resident); Temp: temperature; TL: total length.

Model averaging of cumulative AIC weights supported the model selection, and parameters in the top candidate models for both 50% and 95% KD estimates were shown to influence (w_i_ > 0.5) activity spaces of grey reef sharks tagged at Pohnpei (Table 4). Month was the most influential parameter for the core and extent of activity space, followed by grouper spawning period, with activity spaces contracting during these periods (Fig 4 and Table 4).

**Table 4:** Cumulative AIC weights (w), parameter estimates, standard error (SE), and p-values where deltaAIC < 2.

Parameter	Estimate	SE	p	w_i_
50% KD (km^2^)				
**Month**	**0.0004**	**0.0001**	**0.0004**	**1.00**
**Spawning**	**-0.0033**	**0.0020**	**0.1030**	**0.68**
Temp	0.0022	0.0032	0.4907	0.25
Resident	-0.0032	0.0033	0.3352	0.17
TL	-0.0001	0.0002	0.6963	0.12
95% KD (km^2^)				
**Month**	**0.0720**	**0.0211**	**0.0007**	**1.00**
Spawning	-0.5579	0.3855	0.1493	0.48
Temp	-0.3767	0.6081	0.5368	0.22
Resident	-0.6980	0.8759	0.4272	0.23
TL	-0.0098	0.0585	0.8672	0.09

Cumulative AIC weights (w), parameter estimates, standard error (SE), and p-values from an average subset of candidate models where deltaAIC < 2. Parameters considered influential drivers of activity space (50% and 95% KD) are in bold. Resident: residency status (transient, semi-transient, resident); Temp: temperature; TL: total length.

During aggregation months, RI was lowest and most variable during January when only *P*. *areolatus* is present in the FSA site. Previous work at the FSA site suggests that it is during this period when *P*. *areolatus* aggregations are highly or exclusively composed of males.

### Inter-reef and long-distance movement

In general, grey reef sharks tended to be highly resident to the island or atoll where they were captured, however six females tagged at Pohnpei (35%) were detected at Ant Atoll. Of the four sharks tagged at Ant Atoll, Transmitter 51334 was detected only for one day and subsequently removed from the analysis. Two females were never detected at any station at Pohnpei and the fourth (Transmitter 48010) was detected at Pohnpei for 39 days out of the 318 total detection days, with the remainder of detections at Ant.

## Discussion

In Pohnpei, Micronesia, grouper spawning events appear to influence the movement patterns and activity spaces of semi-transient and transient female grey reef sharks. During the grouper spawning season, grey reef shark activity spaces contracted around FSA sites, daily detections increased and the number of tagged sharks present at the FSA rose, in support of prior research that demonstrated the use of FSA for foraging by elasmobranchs. While the scale of movement and activity space for all sharks extended well beyond MPA boundaries (95% KD range = 2.1–109.0 km^2^; average = 12.0±25.3 km^2^) during non-spawning months, most sharks tagged were detected within the KMS on more than 50% of monitored days. For resident sharks, the small activity space and extensive time within the MPA provides proof that small-scale MPAs have benefits to at least some individuals and offers partial benefits to others. These combined findings highlight that, although limited spatially and temporally, small-scale MPAs have utility in reducing the vulnerability of sharks and groupers to fishing during periods when they are concentrated. Conversely, the large-scale movements and associated activity spaces of sharks outside of grouper spawning times, and the use of common reproductive migratory corridors by groupers, supports a combined approach to management that limits fishing impacts outside of reproductive periods and away from MPAs.

Previous published reports have shown the direct trophic benefits to elasmobranchs from FSA foraging [2–4, 14, 20–21, 23–25, 35]. For example, spawning aggregations of *E*. *polyphekadion* in Fakarava, French Polynesia, and presumably elsewhere, create the conditions for an inverted biomass pyramid that allows grey reef (and possibly other) sharks to maximize foraging success and minimize energetic, long-distance movements during aggregation periods [14]. Although typically less abundant than Fakarava, *E*. *polyphekadion* FSA of similar densities [33, 47] are known throughout the Indo-Pacific [32, 78], many in association with the same (or congeneric) grouper species found in Pohnpei. While predation success rates were recorded as low in Fakarava, the concentration of prey nonetheless increased opportunities for foraging relative to non-reproductive periods [14]. The FSA also appeared to attract sharks from a substantially larger catchment area. Similar foraging events have been observed by whale sharks on cubera snapper (*Lutjanus cyanopterus*) (Cuvier 1828) FSA [4] and whale sharks and bull sharks (*Carcharhinus leucas*) (Muller & Henle 1839) on mutton snapper (*Lutjanus analis*) (Cuvier 1828) spawn and spawners, respectively [79]. Alfred mantas have been observed schooling and feeding on eggs produced within surgeonfish FSA [23]. For other reef and nearshore species, oophagy has been widely reported, while defecation of digested materials provide nutrients to a wide range of pelagic and benthic organisms [2–7]. Clearly, FSA serve as productivity hotspots and have important benefits to ecosystem health and trophodynamics at multiple scales [5–7]. These benefits are expected to increase as FSA biomass and reproductive output increase. In recognition of these food web linkages, FSA protection provides multiple benefits to ecosystems, including sharks and rays.

In Pohnpei, acoustic telemetry surveys clearly suggest that small-scale MPAs can provide ephemeral protection to female *C*. *amblyrhynchos*, with the level of protection varying by shark residency type. Specifically, resident sharks would be expected to receive greater benefits both spatially and temporally from these small set-asides, while for transient and semi-transient sharks, the protections more closely resemble that of aggregating grouper, given the extent of movement and use of common reproductive migratory corridors by these transient spawners. For aggregating groupers, at least some individuals have been detected or captured up to 27 kms from the FSA site [62–64], with pathways typically along outer reef areas to reach home reefs. The scale of movement suggests that catchment areas for groupers are on the order of 100s of km^2^ [63], thus similar to non-resident sharks. For coastal sharks as well as aggregating grouper, large-scale no-entry or no-take management options appear to be more optimal solutions than small MPAs to long-term population persistence. However, the degree of area protection needed is both politically and socially untenable in PICTs where coastal fisheries represent important sources of protein and income, and fishing areas are inherently limited.

Although there is no formal enforcement, the Federated States of Micronesia provided blanket protection for sharks from fishing within the 3-million km^2^ economic exclusion zone (EEZ) in 2015 through the passage of Public Law No. 18–108. Owing to the limited reliance on sharks for either food or income, the law received little opposition. In addition to the FSM shark law, in 2017 Pohnpei State extended its own exclusion zone from 12 to 24 miles (38.6 km) (Public Law 19–167) preventing foreign commercial fishing from nearing its coastlines. Such large-scale MPAs could provide the protection required by these animals, however it is currently unclear how effective enforcement of such large areas can be accomplished.

For grouper FSA, Pohnpei modified its two-month (1 March– 30 April) grouper sales ban to extend protection throughout the spawning season (1 January to 31 May). The law was further amended to extend the sales ban to include catch and possession of groupers during this period. Unfortunately, the law provided a loophole allowing individual fishers to take 10 groupers per person per day, which greatly offsets any gains that would have otherwise been realized. Moreover, extended seasonal bans tend to exert greater fishing pressure on other vulnerable species when fishers’ primary goals are catch volume and not species composition [80]. In contrast, an inclusion of a catch ban to the grouper sales ban may lessen the attractiveness of FSA fishing and thus reduce vulnerability on both groupers and sharks at the aggregation site. Ultimately, an ecosystem-based management plan would benefit these various species and, in particular, be more suitable to both resident and transient sharks.

Sharks are often sex segregated during non-reproductive periods [51, 53, 59] and are reportedly impacted differentially by various types of fishing gear. In Pohnpei, fishing in relatively shallow water (*c*. 30 m) along outer barrier reef walls and slopes predominantly yielded only large females. The paucity of males and juveniles in this (and other) study somewhat limits the conclusions that can be made regarding species-level conservation for grey reef sharks using small-scale MPAs, however it also highlights the need to conduct more robust investigations integrating all life history stages. The studies conducted to date also reveal the complexity associated with shark conservation using small-scale area protection. Reef connectivity, shark development stage, reproduction, parturition and sex clearly have a bearing on reserve design, including placement, area and depth [81]. Future efforts to describe the conservation benefits of small- and large-scale MPAs, especially those focused on FSA areas should, if possible, incorporate all sizes and sexes of target animals utilizing a wider capture area, and use a range of habitats to enable more generalized statements on the design and spatial habitat required for effective nearshore shark conservation. Finally, information on grey shark reproductive periods and locations would benefit our understanding of spatial habitat associations and conservation needs.
